# Short Incubation of Positive Blood Cultures on Solid Media for Species Identification by MALDI-TOF MS: Which Agar Is the Fastest?

**DOI:** 10.1128/spectrum.00038-21

**Published:** 2021-06-09

**Authors:** Neele J. Froböse, Evgeny A. Idelevich, Frieder Schaumburg

**Affiliations:** a Institute of Medical Microbiology, University Hospital Münster, Münster, Germany; b Friedrich Loeffler-Institute of Medical Microbiology, University Medicine Greifswald, Greifswald, Germany; Broad Institute

**Keywords:** blood culture, mass spectrometry, matrix-assisted laser desorption ionization–time of flight, culture media, blood culture

## Abstract

Short incubation of positive blood cultures on solid media is now increasingly applied to speed up species identification by matrix assisted laser desorption ionization–time of flight mass spectrometry (MALDI-TOF MS). Although Columbia blood agar (CBA) and chocolate agar (Choc) are widely used, a direct comparison of standard agars is lacking. We therefore compared the time to species identification of blood cultures incubated on CBA, Choc, and MacConkey agar (MAC, for Gram-negative rods). Positive aerobic/anaerobic blood cultures (2 drops = 50 μl) were incubated on CBA, Choc, MAC, and the required time of incubation to low-confidence identification (score of ≥1.7 to <2) and high-confidence identification (score of ≥2) by MALDI-TOF MS was measured. Exclusion criteria were (i) false-positive blood cultures, (ii) mixed cultures with different species, (iii) growth of anaerobes/fungi, and (iv) a total number of isolates of one group (i.e., Gram-positive/-negative cocci/rods) of <30. A total of 187 blood cultures with Gram-positive cocci (*n* = 124) and Gram-negative rods (*n* = 63) were included in the final analysis. The shortest median time to high-confidence identification (score of ≥2) was achieved on MAC for Gram-negative rods (2.0 h; range, 1.9 to 4.2 h) and on CBA for Gram-positive cocci (4.0 h; range, 1.9 to 25.0 h). However, the difference from results obtained with Choc was not statistically significant. When only one agar plate is used for short incubation of positive blood cultures, Choc may represent a compromise in terms of time to high-confidence identification by MALDI-TOF MS and the bacterial spectrum that is covered. However, using only Choc is disadvantageous when the shortest incubation times to identification are strived for.

**IMPORTANCE** When blood cultures are flagged as positive, they are incubated on solid media to produce enough biomass of the bacterium for identification and susceptibility testing. Rapid turnaround times for laboratory results could save lives, and we wanted to assess which solid medium is best to shorten the time to species identification using MALDI-TOF mass spectrometry. For that purpose, we used positive blood cultures from routine diagnostics and compared Columbia blood agar (CBA), Chocolate agar (Choc), and MacConkey agar (MAC, for Gram-negative rods). We found that MAC performed best for Gram-negative rods and CBA was quickest for Gram-positive cocci. However, Choc may represent a compromise if fastidious species should be covered.

## INTRODUCTION

Blood cultures are key for the diagnosis of bacteremia. Rapid species identification and antimicrobial susceptibility testing are essential for management of patient with bacteremia (1–3). To accelerate species identification, increasing numbers of laboratories (ca. 40% in Europe in 2016-2017) incubate positive blood cultures on solid media for a few hours (e.g., 3 to 6 h) to gain sufficient bacterial mass for species identification by matrix-assisted laser desorption ionization–time-of-flight mass spectrometry (MALDI-TOF MS) (4). This ultrashort incubation is usually done on a single plate if laboratories have to save costs. For that purpose, Columbia blood agar (CBA) or chocolate agar (Choc) is frequently used, but a direct comparison of various standard agars is lacking (5–7). The use of CBA entails the risk of missing fastidious bacteria (e.g., bacteria from the HACEK [Haemophilus, Actinobacillus actinomycetemcomitans, Cardiobacterium hominis, Eikenella corrodens, and *Kingella*] group), which might be overcome by using Choc (8). However, during daily routine diagnostics, our technicians felt that species identification by MALDI-TOF MS takes longer when Choc is used than when CBA is used. This prompted us to compare the incubation times of positive blood cultures on three standard solid media (CBA, Choc, and MacConkey agar [MAC]) until a reliable species identification by MALDI-TOF MS could be achieved.

## RESULTS

A total of 232 blood cultures were eligible between 1 September and 1 November 2020. Of these, 45 blood cultures were excluded *post hoc* due to false-negative blood cultures (*n* = 26), mixed cultures (*n* = 11), growth of obligate anaerobes/fungi (1 Staphylococcus saccharolyticus, 1 Cutibacterium acnes, 2 Fusobacterium nucleatum, *2 Candida* sp.) and a sample size of <30 (2 isolates of Gram-positive rods [*Corynebacterium* sp.]).

Hence, 187 samples were included in the final analysis. The greatest number of Gram-positive cocci (*n* = 124) were coagulase-negative staphylococci (*n* = 63), followed by *Enterococcus* sp. (*n* = 34), Streptococcus sp. (*n* = 17), Staphylococcus aureus (*n* = 6), *Rothia* sp. (*n* = 2), and *Gemella* sp. and *Micrococcus* sp. (*n* = 1 each). Among Gram-negative rods (*n* = 63), Escherichia coli was predominant (*n* = 21), followed by Klebsiella pneumoniae (*n* = 10), Enterobacter cloacae complex (*n* = 10), Klebsiella oxytoca (*n* = 6), Serratia marcescens (*n* = 6), Klebsiella variicola and Proteus mirabilis (*n* = 3 each), Pseudomonas aeruginosa (*n* = 2), and Aggregatibacter actinomycetemcomitans and Stenotrophomonas maltophilia (*n* = 1, each).

All time points of MALDI-TOF measurements for individual blood cultures were within the predefined ranges (i.e., ±10 min [±0.17 h] for the first five measurements). For the statistical comparison of time intervals, we applied two approaches. First, intervals accurate to the minute of species identification were applied to detect even small differences in times. Second, intervals rounded to the nearest hour were applied to control for randomly early or late measurements within the defined time ranges.

The shortest median time until a low-confidence identification score was achieved on MAC for Gram-negative rods (2.0 h) and on CBA for Gram-positive cocci (3.0 h, Table 1). When the agars were compared within the groups of Gram-negative rods and Gram-positive cocci, the times until a low-confidence identification was reached were not statistically significantly different (apart from Gram-positive cocci applying time intervals accurate to the minute; *P* = 0.048) (Table 1).

**TABLE 1:** Shortest incubation times of positive blood cultures on solid media until MALDI-TOF MS species identification^*a*^

Score	Microscopy of positive blood cultures (*n*)	Median incubation time (range) (h) on:			*P* value with time:	
		Columbia blood agar	MacConkey agar	Chocolate agar	Not rounded^*b*^	Rounded^*c*^
≥1.7–<2	Gram-negative rods (63)	2.0 (1.9–2.9)	2.0 (1.9–2.0)	2.0 (1.9–24.0)	0.12^*d*^	0.54^*d*^
	Gram-positive cocci (124)	3.0 (1.9–6.1)	NA	3.08 (1.9–23.6)	0.048^*e*^	0.05^*e*^
	All (187)	3.0 (1.9–6.1)	1.99 (1.92–2.02)	3.00 (1.9–24.0)	ND	ND

≥2	Gram-negative rods (63)	2.1 (1.9–24.0)	2.0 (1.9–4.2)	2.1 (1.9–4.2)	0.21	0.07
	Gram-positive cocci (124)	4.0 (1.9–25.0)	NA	4.2 (2.0–25.8)	0.13	0.13
	All (187)	3.0 (2.0–25.0)	2.0 (1.9–4.2)	3.5 (2.0–25.8)	ND	ND

a NA, not applicable; ND, not done.

b Considering time intervals accurate to the minute.

c Considering time intervals rounded to the nearest hour.

d Kruskal-Wallis rank sum test.

e Wilcoxon rank sum test.

Similar to low-confidence identification, the shortest median time to high-confidence identification (score, ≥2) was achieved on MAC for Gram-negative rods (2.0 h; range, 1.9 to 4.2 h) and on CBA for Gram-positive cocci (4.0 h; range, 1.9 to 25.0 h). The comparison of the different agars within the groups of Gram-negative rods and Gram-positive cocci revealed no significant difference in the times until a high-confidence identification was reached (Table 1).

The proportion of high-confidence identification of Gram-negative rods was significantly higher with MAC than with Choc at 2 h (78% versus 57%; *P* = 0.013, χ^2^ test). For the remaining time points, the proportions were not significantly different in Gram-negative rods (Table 2).

**TABLE 2:** Proportion of positive blood cultures with low- and high-confidence identification at different time points

Score	Time point (h)	Gram-negative rods (*n* = 63)				Gram-positive cocci (*n* = 124)		
		No. (%) with identification on:			*P* value	No. (%) with identification on:		*P* value
		Columbia blood agar	MacConkey agar	Chocolate agar		Columbia blood agar	Chocolate agar	
≥1.7	2	55 (87)	56 (89)	50 (79)	0.3	36 (29.0)	23 (18.5)	0.05
	3	60 (95)	62 (98)	60 (95)	0.5	85 (68.6)	66 (53.2)	0.01
	4	62 (98)	63 (100)	62 (98)	NA	107 (86.3)	92 (74.2)	0.02
	5	62 (98)	63 (100)	62 (98)	NA	116 (93.6)	111 (89.5)	0.25
	6	62 (98)	63 (100)	62 (98)	NA	120 (96.8)	116 (93.6)	0.24
	24	63 (100)	63 (100)	63 (100)	NA	121 (97.6)	120 (96.8)	0.7

≥2	2	41 (65)	49 (78)	36 (57)	0.047	0 (0)	0 (0)	NA
	3	58 (92)	61 (97)	58 (92)	0.45	9 (7.3)	7 (5.7)	0.6
	4	62 (98)	63 (100)	62 (98)	NA	43 (34.7)	33 (26.6)	0.17
	5	62 (98)	63 (100)	62 (98)	NA	81 (65.3)	63 (50.8)	0.02
	6	62 (98)	63 (100)	62 (98)	NA	103 (83.1)	90 (72.6)	0.47
	24	63 (100)	63 (100)	62 (98)	NA	111 (89.5)	106 (85.5)	0.34

For Gram-positive cocci, CBA yielded significantly higher proportions of low-confidence identifications between 3 and 4 h (*P* ≤ 0.02) or high-confidence identification at 5 h (*P* = 0.02) (Table 2) than Choc.

## DISCUSSION

We compared three different standard agars for the ultrashort incubation of blood cultures prior to species identification by MALDI-TOF MS and found that MAC performed best for Gram-negative rods and CBA was quickest for Gram-positive cocci.

The predominance of coagulase-negative staphylococci among Gram-positive cocci and E. coli among Gram-negative rods is in line with other studies and allows a direct comparison of incubation times with these studies (5, 6, 9).

The proportion of high-confidence species identification after 4 to 5 h on CBA was similar to or even better than that in previous studies for Gram-negative rods (98% versus 90.6 to 95.2%) and Gram-positive cocci (65.3% versus 87.9%) (5, 6). Our study indicates that the use of Choc causes a minor delay in the median time to low-confidence identification, but this effect was not detected when the time to high-confidence identification was assessed (Table 2). These differences become obvious only after incubation times of 2 h (Gram-negative rods) or 5 h (Gram-positive cocci), when the proportion of identification scores that are ≥2 is still low (50 to 78%) (Table 2).

Mixed cultures should be excluded when the ultrashort incubation on solid media is applied, as species identification is unreliable or misleading, as shown with urine samples (10). As mixed cultures cannot be ruled out by Gram staining of blood culture smears, (e.g., E. coli and K. pneumoniae are indistinguishable), pure cultures should be confirmed after 18 to 24 h on the applied solid medium.

The selection of agar plates depends on the health care setting and which species are expected in the majority of blood cultures. If pathogens associated with hospital-acquired infections (e.g., *Enterococcus*, Staphylococcus, *Enterobacterales*, and Pseudomonas) dominate the expected spectrum, it could be justified to use only CBA plates for the ultrashort incubation of blood cultures, or MAC may be used if Gram-negative rods are present in the smear. If fastidious species are expected (e.g., HACEK organisms), Choc should be among the selected solid media.

Our study has limitations. First, we did not include a high number of fastidious bacteria and therefore are not able to determine to what extent the use of CBA could accelerate the diagnosis of these pathogens. Second, ultrashort incubation and species identification via MALDI-TOF MS are to some extent dependent on the individual method of picking bacterial colonies and applying them on the target plate and can differ between laboratory technicians.

Although MAC performed best for Gram-negative rods and CBA for Gram-positive cocci, the differences from Choc were only marginal. When only one agar plate is used for short incubation of positive blood cultures, Choc may be a compromise in terms of time to high-confidence identification by MALDI-TOF MS and the bacterial spectrum that is covered. However, using only Choc is disadvantageous when the shortest incubation times to identification are strived for.

## MATERIALS AND METHODS

### Ethics.

Ethical approval was obtained from the institutional review board (IRB, Ethikkommission der Westfälischen Wilhelmsuniversität Münster; 2021-002-f-S). The IRB granted a waiver to obtain a signed written informed consent from patients.

### Sample size calculation.

We were unable to identify studies that could substantiate any assumptions about the difference in incubation time on all test agars. We therefore did not calculate the sample size and deemed a sample of 200 consecutive blood cultures as appropriate for the objective (as used in other protocols investigating ultrashort incubation) (5).

### Culture.

All aerobic/anaerobic blood cultures (Bactec; Becton Dickinson [BD], Heidelberg, Germany) that were flagged as positive in a Bactec 9240 (BD) between 6.30 p.m. and 9.00 a.m. were included. Bottles flagged as positive overnight starting from 6:30 p.m. remained in the Bactec 9240 system and were processed the next morning at 7:30 a.m. This time interval was chosen to complete the following analysis steps within a working day. *Ex ante* exclusion criteria were (i) transport time to the laboratory of >24 h, (ii) bottles filled with nonblood specimens (e.g., pleural effusion or cerebrospinal fluid [CSF]), and (iii) BD Bactec bottles specific for smaller volumes (Peds), fungi (Mycosis), and mycobacteria (Myco/F). *Post hoc* exclusion criteria were (i) false-positive blood cultures, (ii) mixed cultures with different species, (iii) growth of anaerobes/fungi, and (iv) a total number of isolates within one group (i.e., Gram-positive/-negative cocci/rods) of <30.

Positive blood cultures were microscopically examined (Gram stain), and two drops (=50 μl) of blood culture broth was streaked on six plates each (all BD) of CBA, Choc, and MAC (if Gram-negative rods were seen) prior to incubation at 5% CO_2_ and 36 ± 1°C (5). Species identification was done at 2 h, 3 h, 4 h, 5 h, 6 h (±10 min), and 24 h (±6 h) after the start of the incubation on the solid media.

One of the six plates of each agar type for one specific time point was used for MALDI-TOF MS measurement, allowing the remaining plates to be incubated continuously in the meantime. All agar plates were incubated overnight at 5% CO_2_ and 36 ± 1°C prior to the inoculation with positive blood culture broth, so that plates and incubator would have the same temperature when positive blood cultures were applied, and to control plates for sterility.

### MALDI-TOF MS.

After deposition of microbial biomass (the amount that adheres to a sterile toothpick tip) onto a polished steel MALDI target, 1 μl of 70% formic acid was added and allowed to dry. This was followed by overlaying with α-cyano-4-hydroxycinnamic acid matrix and MALDI-TOF MS measurement using the Microflex instrument (Bruker, Bremen, Germany) and MBT Compass software (version 4.1.80; Bruker). Time intervals for each solid medium were recorded until low-confidence identification (score of ≥1.7 to <2) and high-confidence identification (score of ≥2) were achieved. If a score of ≥2 was obtained, the respective colonies were not tested again at later time points.

### Statistics.

As data points were not normally distributed, we applied the Wilcoxon rank sum test for the comparison of two agars (i.e., Gram-positive bacteria) or the Kruskal-Wallis test for the comparison of the three different agars (i.e., applying to Gram-negative rods). The Holm method was done to correct for multiple testing. Agar comparison of all isolates was omitted because only Gram-negative bacteria grow on MAC and they generally grow faster, leading to a systematic overvaluation of MAC. Categorical variables were compared with χ^2^/Fisher’s exact test where appropriate. All analyses were done with R (version 3.6.1, package epiDisplay).
